# Development of a *Bacillus subtilis *expression system using the improved P*glv *promoter

**DOI:** 10.1186/1475-2859-9-55

**Published:** 2010-07-10

**Authors:** Yang M Ming, Zhang W Wei, Chen Y Lin, Gong Y Sheng

**Affiliations:** 1 College of Animal Science and Technology, Northwest A&F University, Yangling 712100, China

## Abstract

**Background:**

*B. subtilis *is an important organism in the biotechnological application. The efficient expression system is desirable in production of recombinant gene products in *B. subtilis*. Recently, we developed a new inducible expression system in *B. subtilis*, which directed by *B. subtilis *maltose utilization operon promoter P*_glv_*. The system demonstrated high-level expression for target proteins in *B. subtilis *when induced by maltose. However, the system was markedly repressed by glucose. This limited the application of the system as a high-expression tool in biotechnology field. The aim of this study was to further improve the P*_glv _*promoter system and enhance its expression strength.

**Results:**

Here, site-directed mutagenesis was facilitated to enhance the expression strength of P*_glv_*. The transcription level from four mutants was increased. Production of β-Gal from the mutants reached the maximum 1.8 times as high as that of wildtype promoter. When induced by 5% maltose, the production of β-Gal from two mutants reached 14.3 U/ml and 13.8 U/ml, 63.5% and 57.5% higher than wildtype promoter (8.8 U/ml) respectively. Thus, site-directed mutagenesis alleviated the repression of glucose and improved the expression activity. To further improve the promoter system, the *B. subtilis *expression host was reconstructed, in which *B. subtilis *well-characterized constitutive promoter P43 replaced the promoter of the *glv *operon in *B. subtilis *chromosome through a double crossover event. The β-galactosidase production from the improved system (21.1 U/mL) increased compared to that from origin system. Meanwhile, the repression caused by glucose was further alleviated.

**Conclusions:**

In this study, we obtained a mutated promoter P*glv*-M1 through site-directed mutagenesis, which demonstrated high expression strength and alleviated the repression caused by glucose. Moreover, we alleviated the repression and enhanced the expression activity of the P*glv*-M1 promoter system via reconstruction of the *B. subtilis *host. Thus, we provided a valuable expression system in *B. subtilis*.

## Background

*B. subtilis *is an important organism in the biotechnological application, regarding its non-pathogenic and well-characterized biochemical and physiological property [1,2]. The genetic engineering of *B. subtilis *played a significant role in biotechnological application and industry [3,4]. A controllable expression system is desirable in efficient production of recombinant gene products in *B. subtilis *[5-7]. The most prominently and widely used induction systems in *B. subtilis *are mediated by promoters P_spac _and P*_xyl_*. The disadvantage of these systems is that the inducer is costly for industrial application [8-10].

Recently, we developed a new inducible expression system in *B. subtilis *[11], which directed by *B. subtilis *maltose utilization operon promoter P*_glv _*[12,13]. The system demonstrated high-level expression for target proteins in *B. subtilis *when induced by maltose. Moreover, cheap and safe inducer makes the system a potential promoter system in industrial application. However, the system was markedly repressed by glucose, in which the glucose repressed the P*_glv _*promoter via a catabolism repression element (*cre*) located downstream of the transcription origin site of the P*_glv _*promoter [11-13]. This limited the application of the system as a high-expression tool in biotechnology field. To improve the P*_glv _*promoter system, site-directed mutagenesis of several nucleotides downstream the transcription origin site of P*_glv _*was facilitated via overlap polymerase chain reaction (PCR) *in vitro *in this study. To further alleviate the repression and enhance the expression strength of the P*_glv _*promoter, the *B. subtilis *expression host was reconstructed, in which the constitutive promoter P43 replaced the promoter of the *glv *operon in the *B. subtilis *chromosome through a double crossover event.

## Results and discussion

### Site-direct mutagenesis of P*glv *promoter and examination of the expression strength

In the mutant *P_glv_*-M1, two bases located at the conservation sequence of catabolism repression element were mutated (GC→AT). According to the 3' sequence of 16 s RNA, the ribosome binding site was optimized in the mutants *P_glv_*-M2 and *P_glv_*-M3, based on *P_glv_*-M1, Additionally, based on *P_glv_*-M3, the two bases downstream Shine-Dalgarno sequence (SD sequence) of the *P_glv _*promoter was mutated, resulting in *P_glv_*-M4.

There is a typical conservation sequence of catabolism repression element (*cre*), overlapping with the ribosome binding site, downstream of the transcription origin site of the P*_glv _*promoter [11,13]. In order to alleviate the repression of glucose and improve the expression strength of the P*_glv _*promoter, the site-directed mutagenesis of *cre *sequence is performed. To further enhance the expression of gene downstream the promoter, we try to improve the ribosome-binding site sequence. According to the sequence of hydroxyl end of 16 S rRNA, the nucleotides downstream the SD sequence were select to modify in pJRINM4.

**Figure 1:**
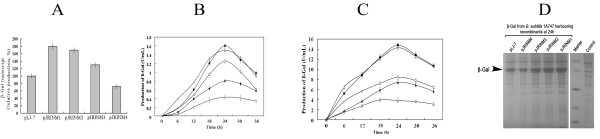
**RT-PCR analysis of the mutated promoters (A) and the production of β-Gal driven by these promoters (B, C and D)**. (A) Real-time PCR analysis of the transcription amount from pLJ-7, pJRINM1, pJRINM2, pJRINM3 and pJRINM4, respectively (16 s rDNA used as the control). (B) The production of β-Gal when cultured in LB. The black square, white triangle, white circle, black diamond and white diamond represents the production of β-Gal from *B. subtilis *1A747 harboring pJRINM1, pJRINM2, pJRINM3, pLJ-7 and pJRINM4, respectively. (C) The production of β-Gal when cultured in LB supplemented with 5% maltose. The black square, white triangle, white circle, black diamond and white diamond represents the production of β-Gal from *B. subtilis *1A747 harboring pJRINM1, pJRINM2, pJRINM3, pLJ-7 and pJRINM4; (D) SDS-PAGE analysis of β-Gal from the recombinants in *B. subtilis *1A747 after 24 h cultured on a 12% SDS-polyacrylamide gel. β-Gal is indicated by the arrow. Molecular mass marker indicates (top to bottom): 116, 66, 45, 35 and 25 kDa.

To examine the expression efficiency of the obtained four mutants, they were sub-cloned and engineered with synthetic ribosome binding site. The resultant recombinants pJRINM1, pJRINM2, pJRINM3 and pJRINM4, in which the *bgaB *was under the control of four mutants respectively, were transformed into *B. subtilis *1A747 to investigate the expression of β-Gal. Real-time PCR assay (Figure 1A) showed that compared with the pLJ-7, the transcription amount from the pJRINM1, pJRINM2 and pJRINM3 were increased in different degrees, in which the mutant pJRINM1 is obviously prior to the pLJ-7. This suggested the site-directed mutagenesis of the Pglv promoter is efficient in these three mutants.

To further verify the efficiency of mutated promoters, the β-Gal driven by these promoters was determined. The production of β-Gal from pJRINM1, pJRINM2 and pJRINM3 increased compared with that from pLJ-7. Amongst the production of β-Gal from pJRINM1, pJRINM2 was 1.8 fold (1.6 U/mL) and 1.7 fold (1.5 U/mL) of that from wildtype promoter (0.9 U/mL) after 24 h culture, respectively. Whereas, the production of β-Gal from pJRINM4 was obviously decreased, about 58.2% of that from wildtype promoter after 24 h culture (Figure 1B). This showed that site-directed mutagenesis in pJRINM4 has negative effect on the P*_glv _*promoter. We speculated that the three mutated sites including the SD sequnence may change the space structure of the promoter, and the result reduced the transcript level.

While, the growth trend of *B. subtilis *1A747 harbouring pJRINM1, pJRINM2, pJRINM3, pJRINM4 or pLJ-7 was approximately same (Additional file 1). It confirmed that the difference of β-Gal production was not caused by the cell amount.

To further assay these promoters, we tested the growth curves (Additional file 2) and the β-Gal activity (Figure 1C) under the induction conditions. When induced with 5% maltose, the production of β-Gal from pJRINM1 and pJRINM2 reached 14.3 U/mL and 13.8 U/ml, up by 63.5% and 57.5% compared with wildtype promoter (8.77U/mL) at 24 h, respectively. Meanwhile, the β-Gal production from pJRINM4 was 4.3 U/mL, only 48.8% of that from wildtype promoter.

SDS-PAGE assay (Figure 1D) demonstrated the β-Gal production from pJRINM1 and pJRINM2 was obviously higher than that from pLJ-7, further confirming that the two mutants enhanced the expression strength of P*_glv _*promoter.

### Effect of site-directed mutagenesis on the repression caused by glucose

**Figure 2:**
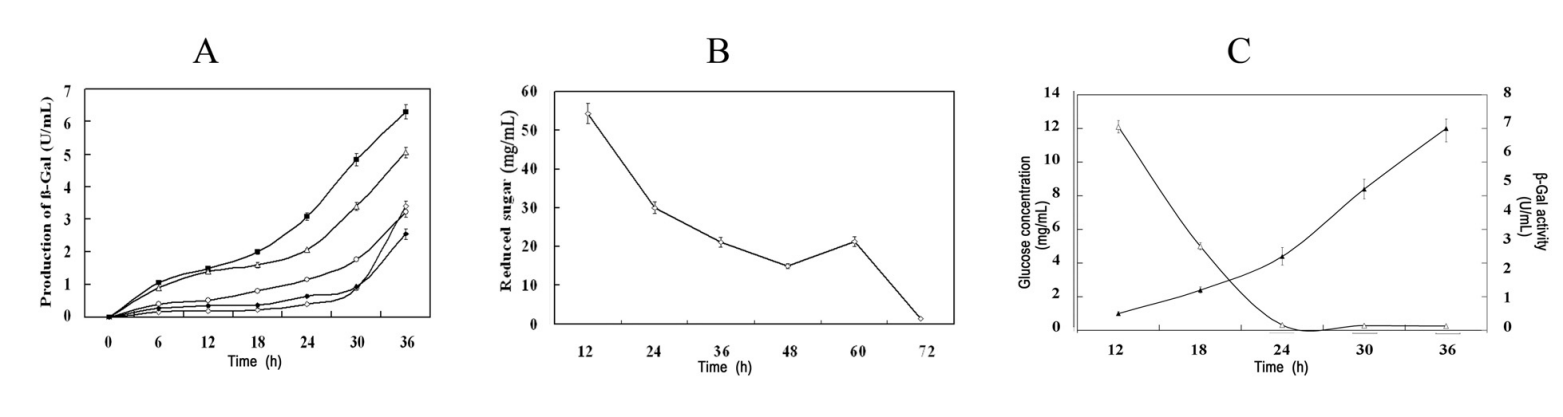
**Characterization of the mutated promoters**. The production of β-Gal from *B. subtilis *1A747 harboring mutants supplemented with 5% maltose plus 5% glucose. The black square, white triangle, white circle, black diamond and white diamond represents the production of β-Gal from *B. subtilis *1A747 harboring pJRINM1, pJRINM2, pJRINM3, pLJ-7 and pJRINM4, respectively; (B) Reduced sugar concentration of culture of *B. subtilis *1A747 harboring pJRINM1 in LB supplemented with 5% maltose; (C) Glucose actual concentration and β-Gal activity detection from *B. subtilis *1A747 harboring pJRINM1 in LB supplemented with 5% maltose plus 5% glucose. The black triangle represents the β-Gal production. The white triangle represents the glucose actual concentration.

To examine the effect of mutant on alleviating repression caused by glucose, the four recombinants were cultured in LB medium supplemented with 5% maltose and 5% glucose, and the growth curves (Additional file 3) and β-Gal production (Figure 2A) was measured, respectively. The β-Gal production from wildtype promoter was only 1/17 and 1/5 of that in medium without glucose supplement at 24 h and 30 h, respectively. Whereas the β-Gal production from pJRINM1 was 1/4 and 1/3 of that in medium without glucose supplement at 24 h and 30 h, respectively. While, the β-Gal production from pJRINM2, pJRINM3 and pJRINM4 was 1/6, 1/6 and 1/4 of that in medium without glucose supplement at 24 h, respectively. Thus, these mutants alleviated the repression caused by glucose, especially pJRINM1.

Taken together, since the pJRINM1 demonstrated advantage in improvement of expression strength and alleviation of repression caused by glucose, the mutated promoter Pglv-M1 is a good candidate as an expression element in *B. subtilis *biotechnology application. Subsequently, an effort was made to further probe the relation between glucose and β-Gal driven by the pJRINM1. When *B. subtilis *harbouring pJRINM1 was cultured in LB medium with 5% glucose, reducing sugar assay (Figure 2B) showed that the reducing sugar decreased from 12 h to 48 h. Additionally, we detected the actual concentration of glucose during the culture. Figure 2C showed a trend that with the decrease of the glucose concentration during cultivation, the β-Gal production from the recombinant sharply increased in the medium supplemented with 5% maltose plus 5% glucose.

### Reconstruction of the *B. subtilis *strain to further improve the expression system

**Figure 3:**
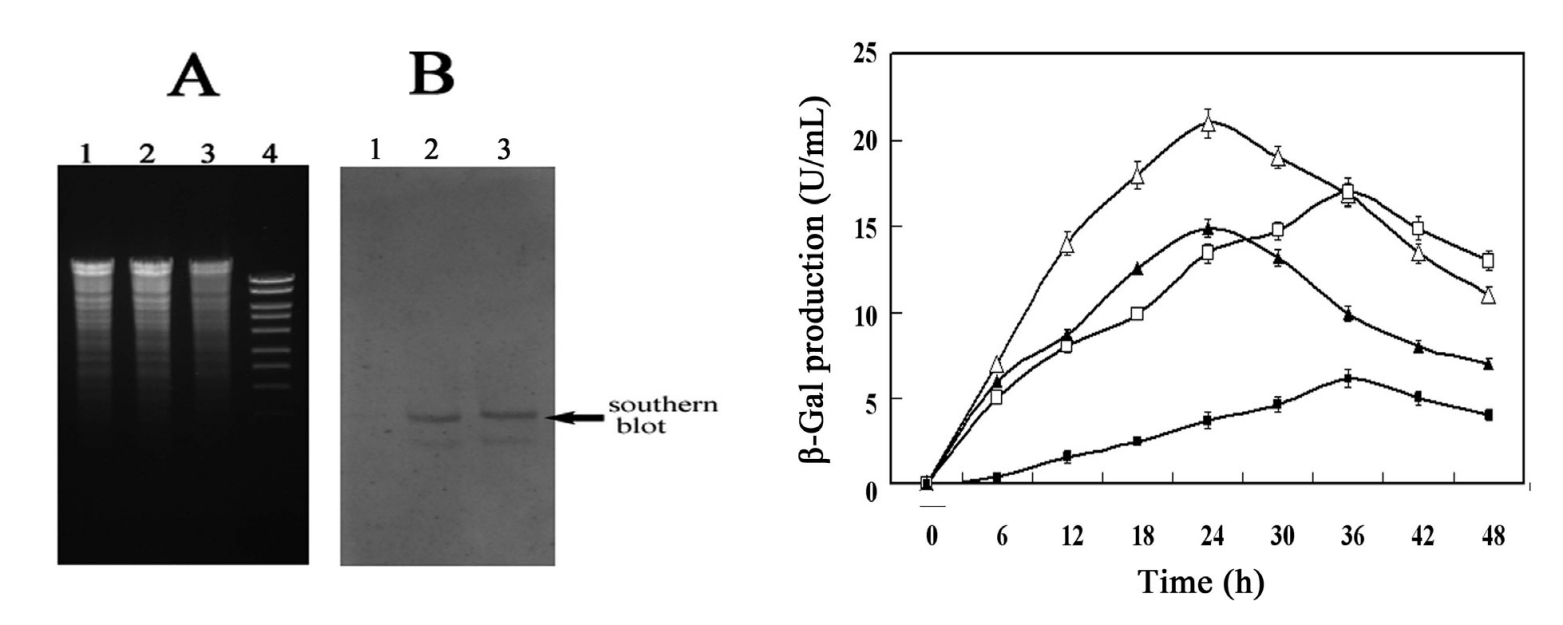
**Reconstruction of *B. subtilis *host**. (A) Agarose-gel-electrophoresis of chromosome DNA digested with *Apa*I and *Eco*RI. Lane1, treated chromosome DNA from *B. subtilis *1A747 [this served as the negative control]; lanes 2 and 3, treated chromosome DNA from *B. subtilis *BCYL; lane 4, molecular weigh marker. (B) Southern blot analysis of recombinant *B. subtilis *BCYL chromosome DNA. The lanes 1, 2 and 3 are that the same as in (A). (C) β-Gal production by the improved expression system. White triangle and white square represents the β-Gal production by *B. subtilis *BCYL harboring pJRINM1 cultures in LB with 5% maltose and LB with 5% maltose plus 5% glucose, respectively; black triangle and black square represents the β-Gal production by *B. subtilis *1A747 harboring pJRINM1 when cultured in LB with 5% maltose and LB with 5% maltose plus 5% glucose, respectively.

The abovementioned results suggested that the promoter P*glv*-M1 alleviated the repression caused by glucose; however, the glucose still exerted repression on the system. We speculated that one of the possible causes of the residual repression was that the maltose utilization operon located in the chromosome of the *B. subtilis *was nevertheless negatively regulated by glucose, in which GlvR as a positive regulator of the Pglv-M1 promoter system was driven by the operon native promoter P*_glv_*. To further improve the Pglv-M1 promoter system, we replaced maltose utilization operon promoter P*glv *with *B. subtilis *constitutive promoter P43. Southern blot analysis (Figure 3A) and PCR detection indicated that the double crossover event occurred in the resultant strain *B. subtilis *BCYL as expected.

To probe the efficiency of the improved expression system, the mutant pJRINM1 was transformed into BCYL and expression experiments were carried out. Figure 3B indicates that, with 5% maltose induction, β-Gal production reached a maximum (21.1 U/mL) at 24 h from *B. subtilis *BCYL harboring pJRINM1, and this was 50.3% higher than that from *B. subtilis *1A747 harboring pJRINM1. For investigating the repression caused by glucose on the improved expression system, BCYL harboring pJRINM1 was cultivated in LB supplemented with 5% maltose plus 5% glucose, yielded about 4-fold higher production of β-Gal than that of *B. subtilis *1A747 harboring pJRINM1 at 24 h (Figure 3B). Therefore, the reporter gene production driven by the improved expression system increased obviously when supplemented with 5% glucose or not.

Controllable and strong promoter is the essential element to achieve high-level expression of target gene in the *B. subtilis *genetic engineering. *B. subtilis *maltose utilization promoter is a potential control element in the biotechnological application; however, glucose as a repressor influenced the strength of P*_glv_*. In the maltose-inducible system, maltose plays two roles as an inductor: on one hand, it positively regulates the transcription of the P*_glv _*promoter; on the other hand, as a fermentation carbon source, its metabolism production-glucose repressed the activity of promoter. There is a dynamic balance between the two carbon sources during the fermentation. Maltose was a positive regulation factor and, meanwhile degraded as one of carbon sources during this process; in the later stage of fermentation, both the total carbon sources and glucose are at relatively low level. When induced by 5% maltose, the production of β-Gal reached the maximum at 24 h and then decreased. In the glucose repression experiment, expression strength of both the mutated and wildtype promoter had a drastic increase after cultured for 24 h. This may be the utilization of glucose, as an easy ferment carbon source, was prior to maltose and the amount of glucose in total carbon source declines to a relatively low level in the later stage of fermentation. As a result, the repression caused by glucose was relatively alleviated after 24 h.

## Conclusions

In this study, we obtained a mutated promoter Pglv-M1 through site-directed mutagenesis, which demonstrated high expression strength and alleviated the repression caused by glucose. Moreover, we further alleviate the repression and enhance the expression activity of the Pglv-M1 promoter system via reconstruction of the *B. subtilis *host. Thus, we improved the promoter system and provided a valuable expression system in *B. subtilis*.

## Methods

### Bacterial strains, plasmids and growth conditions

*B. subtilis *1A747 was a generous gift from the Bacillus Genetic Stock Center (BGSC). *Escherichia coli *DH5α was purchased from Novagen (Darmstadt, Germany). The bacterial strains were cultured in Luria-Bertani (LB) medium at 37°C. Maltose and glucose were added as required. The following concentrations of antibiotics were used for selection: 100 μg/mL ampicillin (Amp), 5 μg/mL chloramphenicol (Cm) and 50 μg/mL spectinomycin (Spec). The plasmids used in this study were listed in Additional file 4.

### Primers and oligonucleotides

Polymerase chain reaction (PCR) primers and oligonucleotides used in this study were listed in Additional file 5.

### General manipulation

General recombinant DNA technique was carried out using standard techniques [14]. The transformation of *B. subtilis *was performed by electroporation [15,16].

### Site-directed mutagenesis of Pglv promoter via overlap PCR

**Figure 4:**
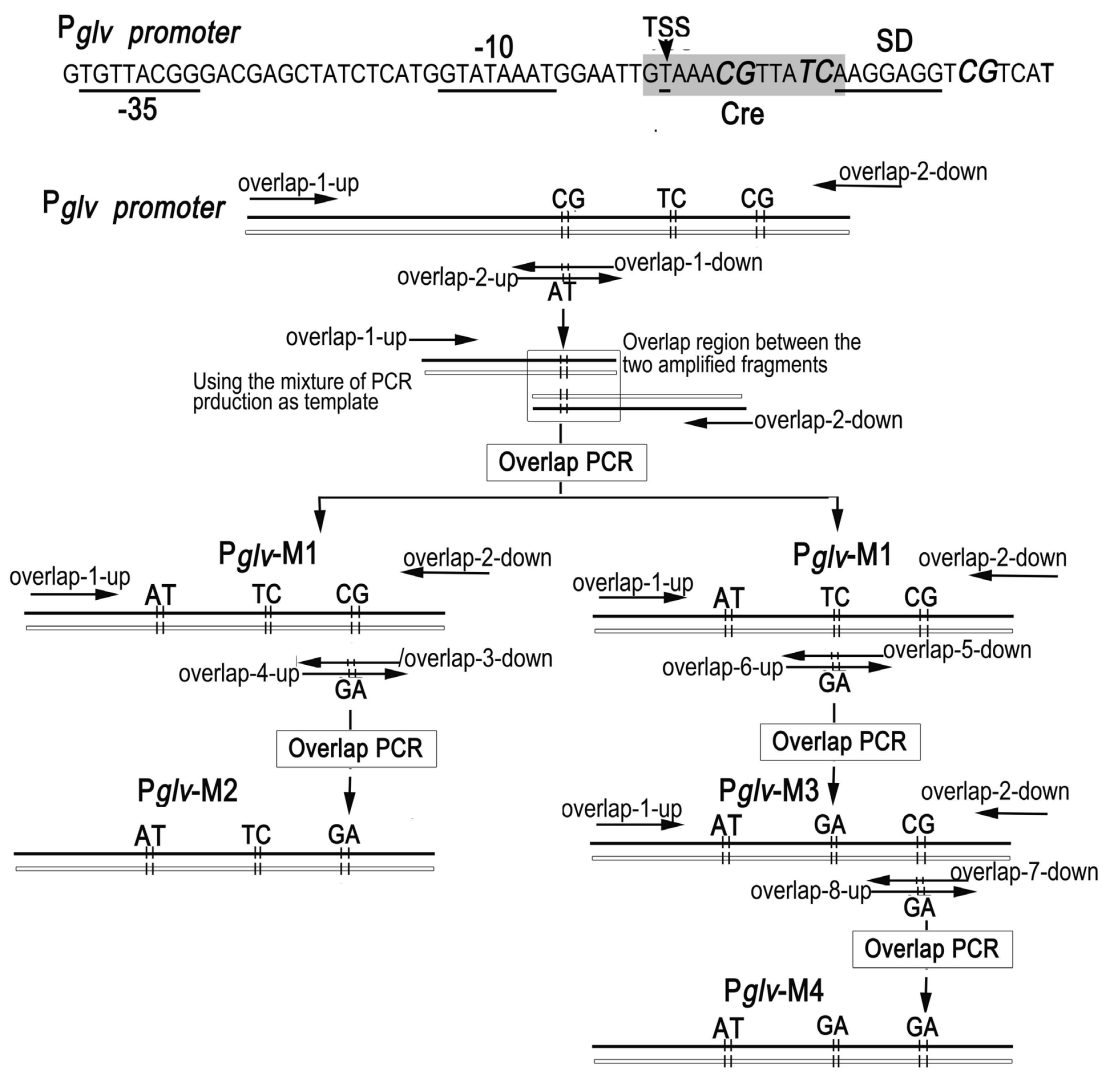
**Schematic diagram of the overlap PCR**. **-**10, -35, TSS, SD, and Cre represent the -10 box, -35 box, transcription origin, SD box and catabolite repression element, respectively. Italic indicates the mutated nucleotides. Approximate positions of the primers are shown with arrows. Using primer pairs overlap-1-up/overlap-1-down and overlap-2-up/overlap-2-down, a 360 bp fragment upstream of the maltose promoter and a 300 bp fragment downstream of the maltose promoter were amplified from *Bacillus subtilis *1A747 genome DNA, in which there are 30 bp overlap in the two amplified fragments and the two mutant sites were introduced by primers overlap-1-down and overlap-2-up. With overlap-1-up and overlap-2-down as primers, and the mixture of two obtained fragments as template, a 630 bp P*_glv_*-M1 was overlap PCR amplified. Same mechanism was used to generate the other three mutants P*_glv_*-M2, P*_glv_*-M3 and P*_glv_*-M4.

The bases downstream the transcription original sites were site-directed mutated by using of overlap PCR. The sequences of primers that were used in this experiment are shown in Additional file 5, and the mechanism of the mutagenesis method and the positions of the primers are depicted in Figure 4.

A 360 bp fragment upstream of the maltose promoter and a 300 bp fragment downstream of the maltose promoter waiting for site-directed mutagenesis were amplified from *Bacillus subtilis *1A747 genome DNA as a template respectively, using primer pairs overlap-1-up/overlap-1-down and overlap-2-up/overlap-2-down, in which there are 30 bp overlap in the two amplified fragments and the two mutant sites were introduced by primers overlap-1-down and overlap-2-up. With overlap-1-up and overlap-2-down as primers, and the mixture of two fragments as template, a 630 bp P*_glv_*-M1 was overlap PCR amplified.

According to the abovementioned protocol, using P*_glv_*-M1 as template, the second mutant P*_glv_*-M2 and third mutant P*_glv_*-M3 were amplified via overlap PCR by using of primer pairs overlap-1-up/overlap-3-down and overlap-4-up/overlap-2-down, and overlap-1-up/overlap-5-down and overlap-6-up/overlap-2-down, respectively. The mutant was introduced by overlap section of overlap-3-down and overlap-4-up for P*_glv_*-M2 and, overlap-5-down and overlap-6-up for P*_glv_*-M3. Then, the fourth mutant P*_glv_*-M4 was generated from P*_glv_*-M3 using primer pairs overlap-1-up/overlap-7-down and overlap-8-up/overlap-2-down, in which the overlap section of overlap-7-down and overlap-8-up introduced the mutant.

### Construction of plasmid vectors

**Figure 5:**
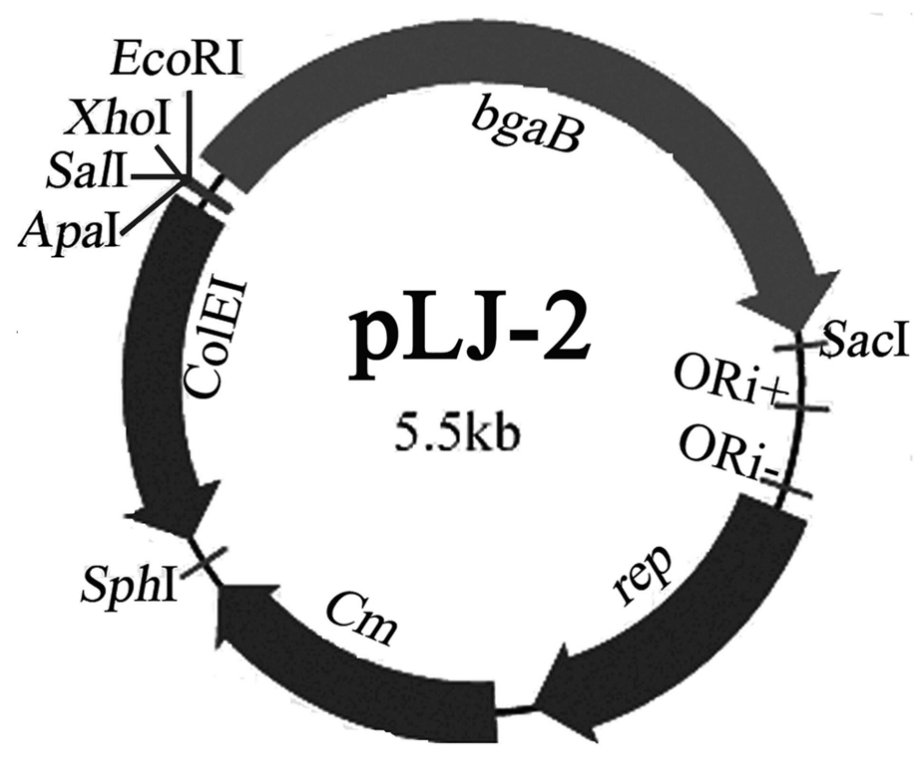
**Map of the promoter probe vector pLJ-2**. ORI+, ORI- and rep represent the single-strand replication origin, the double strand origin and replication protein in ***B. subtilis*, respectively**. ColEI, *bgaB *and Cm represent *E. coli *ColEI replicon, chloramphenicol-resistance marker and coding gene of β-Gal. The unique restriction sites are marked on the outside of the map.

Using primer pair bga-up/bga-down, the *bgaB *coding for thermostable β-Gal was polymerase chain reaction (PCR) amplified from plasmid pDL. The obtained 2.0 kb fragment was digested with *Eco*RI and *Sac*I, and cloned into pGJ103 digested with the same enzymes, resulting in pLJ-2 (Figure 5).

Using Pglv-1-up and Pglv-5-down as primers, P*_glv_*Ma, P*_glv_*Mb, P*_glv_*Mc and P*_glv_*Md were PCR amplified from P*_glv_*-M1, P*_glv_*-M2, P*_glv_*-M3 and P*_glv_*-M4 (As shown in Figure 4), respectively. After digested with *Apa*I and *Bam*HI, these amplified fragments were cloned into the corresponding sites of pBluskm, resulting in pJR1, pJR2, pJR3 and pJR4. A ribosome binding site (RBS) fragment INSD generated by annealing two artificially synthesized oligonucleotides INSID-1 and INSID-2, was cloned into the pJR1-4 digested by *Bam*HI and *Sac*I, respectively. Then, the four mutant promoters with engineered RBS were excised from the obtained four recombinants with *Apa*I and *Eco*RI, and cloned into the corresponding sites of pLJ-2, in which the *bgaB *was used as reporter, yielding pJRINM1, pJRINM2, pJRINM3 and pJRINM4, respectively.

### Reconstruction of *B. subtilis *strain

With *B. subtilis *1A747 chromosomal DNA as template, the two homogeneous arms GAf (using GlvA-fro-up and GlvA-fro-down as primers) and GAb (using GlvA-bac-up and GlvA-bac-down as primers) were amplified. A selection marker (spectinomycin resistance gene) was generated through PCR amplified from plasmid pDG1728, using primers Spec-I-up and Spec-I-down. And *Bam*HI-*Sal*I treated selection marker and *Kpn*I-*Apa*I treated GAf was cloned into pBluskm, resulting in pYG34.

The expression cassette directed by P43 promoter was constructed as follows. First, the P43 promoter [3,17] was amplified from *B. subtilis *1A747 chromosomal DNA, using primers P43-1-up and P43-1-down. After digestion with *Apa*I and *Eco*RI, the P43 promoter was cloned into the corresponding sites in pBluskm, yielding pB43. Then the GAb fragment, digested with *Eco*RI and *Sac*I, was cloned into the corresponding sites in pB43, resulting in pCYL17. Finally, with primers P43-2-up and GlvA-bac-down, the P43 promoter and the GAb were amplified as a single product from pCYL17 and ligated with pYG34 after digestion with *Bam*HI and *Sac*I, resulting in pCYL25. The *B. subtilis *1A747 was transformed with the linear fragment of pCYL25 obtained with *Sca*I to replace promoter P*glv *with P43 via a double crossover event between the linearized pCYL25 and *B. subtilis *1A747 chromosome. The spectinomycin resistance (Spec^R^) colonies were selected. And the resultant strain was named as BCYL.

### Isolation of total RNA and Real-time PCR

The cultures was harvested at 24 h. Total RNA of bacteria was isolated by using SV total RNA isolation kit (Cat. Z3100, Promega). The cDNA chain was synthesized by using Reverse Transcription System (Cat. A3500, Promega).

And Real-time PCR was performed by using Real time PCR Kit (Cat. DRR041 S, TaKaRa). The bagB gene was amplified using bga-up and bga-down as primers B1-up/B1-down. With the primers 16s-up/16s-down, *Bacillus subtilis *16 s rDNA was amplified as control, The PCR protocol was as follows: 2 min at 50°C, 10 min at 95°C, and then 35 cycles consisting of 45 s at 95°C, 1 min at 52°C, and 30 s at 72°C. Reactions were carried out in real-time PCR detection system (IQ5, Bio-RAD).

### SDS-PAGE assay

Sodium dodecylsulfate polyacrylamide gel electrophoresis (SDS-PAGE) was performed as described previously [14].

### β-Gal activity assay

Method has been described previously [[11,18] and [19]]. In brief, the culture was pelleted by centrifuge and resuspended in an equivalent volume of buffer Z [18,19]. Using buffer Z, 0.01 or 0.1 mL sample aliquots was diluted to 0.8 mL, and then added 0.01 mL of lysozyme stock (10 mg/ml). The mixture was incubated at 37°C for 30 min, and then, added 0.2 ml of 4 mg/ml o-nitrophenyl-b-Dgalactopyranoside (ONPG). After incubated at 55°C for 15 min, the reaction was stopped by adding 0.5 ml of 1 M Na_2_CO_3_. Absorbance was recorded at 420 nm with a spectrophotometer (HITACHI, U-3010). One unit of β-galactosidase activity was defined as the amount of enzyme necessary to release 1 μmol 2-nitrophenol from o-nitrophenylgalactopyranoside per minute at 55°C. β-galactosidase activity is expressed as units per mL sample.

### Southern blot analysis

Southern blot analysis of chromosomal DNA digested with *Apa*I and *Eco*RI was carried out as described previously [14]. Probe labeling was performed with a DIG DNA labeling kit (Roche, Cat.No.1093657) according to the instructions, using the spectinomycin resistance gene as template.

### Reducing sugar assay

Reducing sugar was measured by the dinitrosalicylic method (DNS) [20] using glucose as a standard.

### Assay of glucose concentration

The concentration of glucose was determinate as previously described [21]

## Competing interests

The authors declare that they have no competing interests.

## Authors' contributions

MMY initiated and coordinated the project. WWZ and MMY were responsible for site-directed mutagenesis and its analysis. WWZ and YLC performed the batch cultivation. MMY and Y-S Gong performed construction of expression system. All authors wrote the paper and approved the final version of the manuscript.

## Supplementary Material

Additional file 1 **The growth curves from *B. subtilis *1A747 harboring different plasmids when cultured in LB**. (black diamond) represents OD595 from *B. subtilis *1A747 harboring pLJ-7; (black square) represents OD595 from *B. subtilis *1A747 harboring pJRINM1; (black triangle) represents OD595 from *B. subtilis *1A747 harboring pJRINM2; cross (x) represents OD595 from *B. subtilis *1A747 harboring pJRINM3; asterisk (*) represents OD595 from *B. subtilis *1A747 harboring pJRINM4. Click here for file

Additional file 2 **The growth curves from *B. subtilis *1A747 harboring different plasmids when cultured in LB supplemented with 5% maltose**. (black diamond) represents OD595 from *B. subtilis *1A747 harboring pLJ-7; (black square) represents OD595 from *B. subtilis *1A747 harboring pJRINM1; (black triangle) represents OD595 from *B. subtilis *1A747 harboring pJRINM2; cross (×) represents OD595 from *B. subtilis *1A747 harboring pJRINM3; asterisk (*) represents OD595 from *B. subtilis *1A747 harboring pJRINM4. Click here for file

Additional file 3 **The growth curves from B. subtilis 1A747 harboring different plasmids when cultured in LB supplemented with 5% maltose plus 5% glucose**. (black diamond) represents OD595 from *B. subtilis *1A747 harboring pLJ-7; (black square) represents OD595 from *B. subtilis *1A747 harboring pJRINM1; (black triangle) represents OD595 from *B. subtilis *1A747 harboring pJRINM2; cross (×) represents OD595 from *B. subtilis *1A747 harboring pJRINM3; asterisk (*) represents OD595 from *B. subtilis *1A747 harboring pJRINM4. Click here for file

Additional file 4 **Plasmids used in this study**. Click here for file

Additional file 5 **Primers and oligonucletides used in this study**. Click here for file
