# Supplementary figures and images for: Development of a Bacillus subtilis expression system using the improved Pglv promoter
Source: Microb Cell Fact. 2010 Jul 10;9:55. doi: 10.1186/1475-2859-9-55 (PMC2908567; doi:10.1186/1475-2859-9-55)

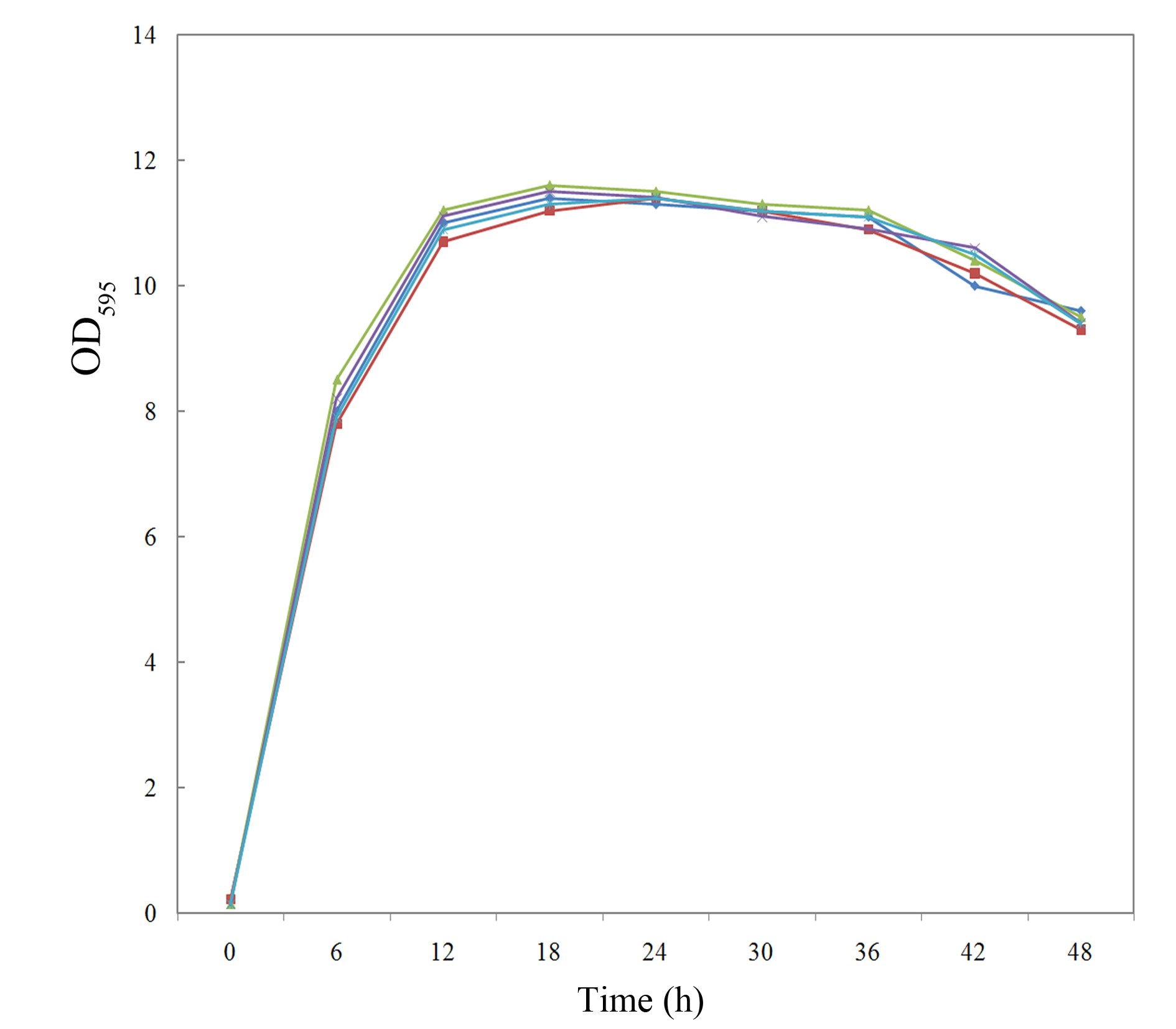

Supplement: Additional file 1 — The growth curves from B. subtilis 1A747 harboring different plasmids when cultured in LB. (black diamond) represents OD595 from B. subtilis 1A747 harboring pLJ-7; (black square) represents OD595 from B. subtilis 1A747 harboring pJRINM1; (black triangle) represents OD595 from B. subtilis 1A747 harboring pJRINM2; cross (x) represents OD595 from B. subtilis 1A747 harboring pJRINM3; asterisk (*) represents OD595 from B. subtilis 1A747 harboring pJRINM4. [file 1475-2859-9-55-S1.TIFF]

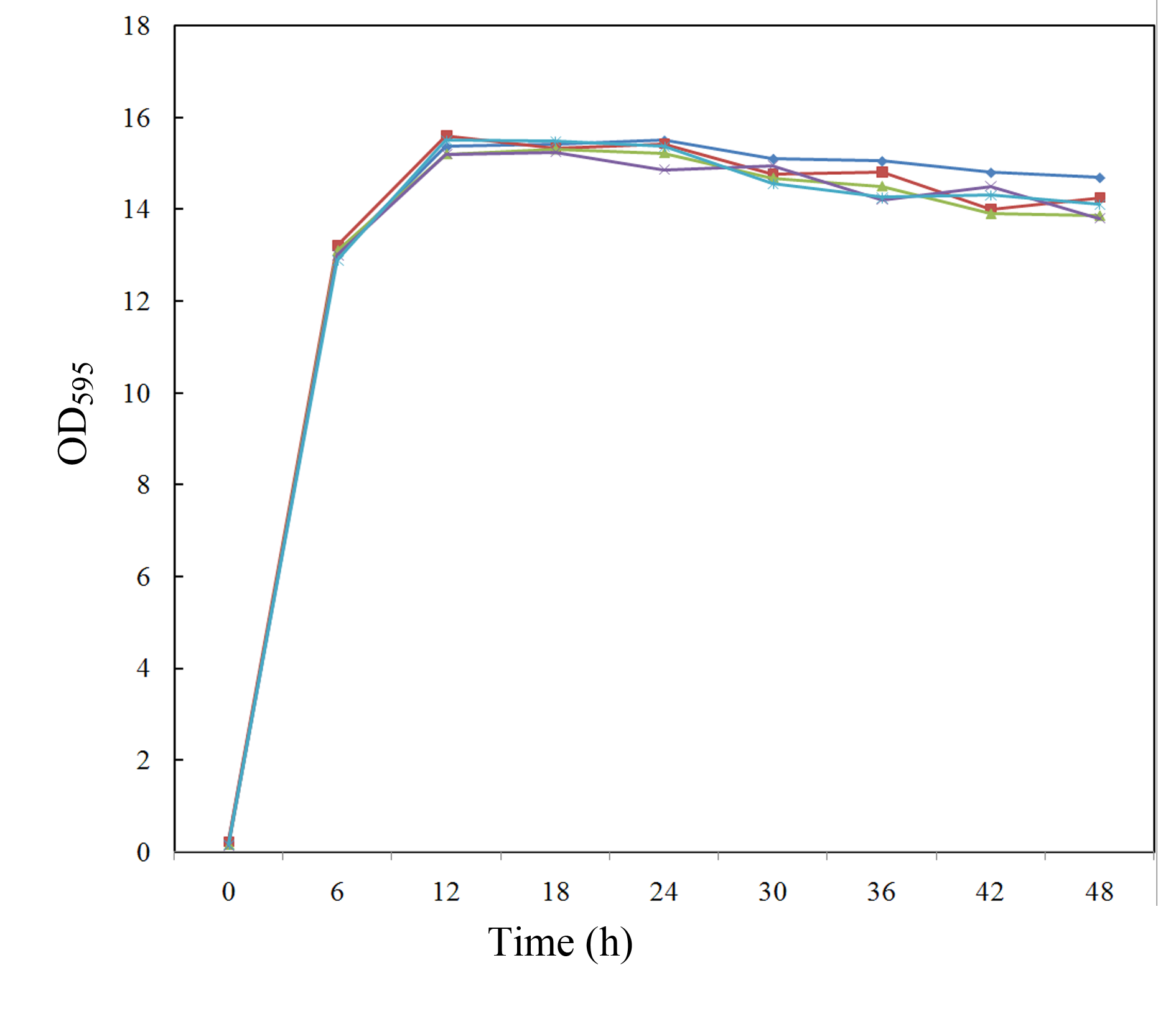

Supplement: Additional file 2 — The growth curves from B. subtilis 1A747 harboring different plasmids when cultured in LB supplemented with 5% maltose. (black diamond) represents OD595 from B. subtilis 1A747 harboring pLJ-7; (black square) represents OD595 from B. subtilis 1A747 harboring pJRINM1; (black triangle) represents OD595 from B. subtilis 1A747 harboring pJRINM2; cross (×) represents OD595 from B. subtilis 1A747 harboring pJRINM3; asterisk (*) represents OD595 from B. subtilis 1A747 harboring pJRINM4. [file 1475-2859-9-55-S2.TIFF]

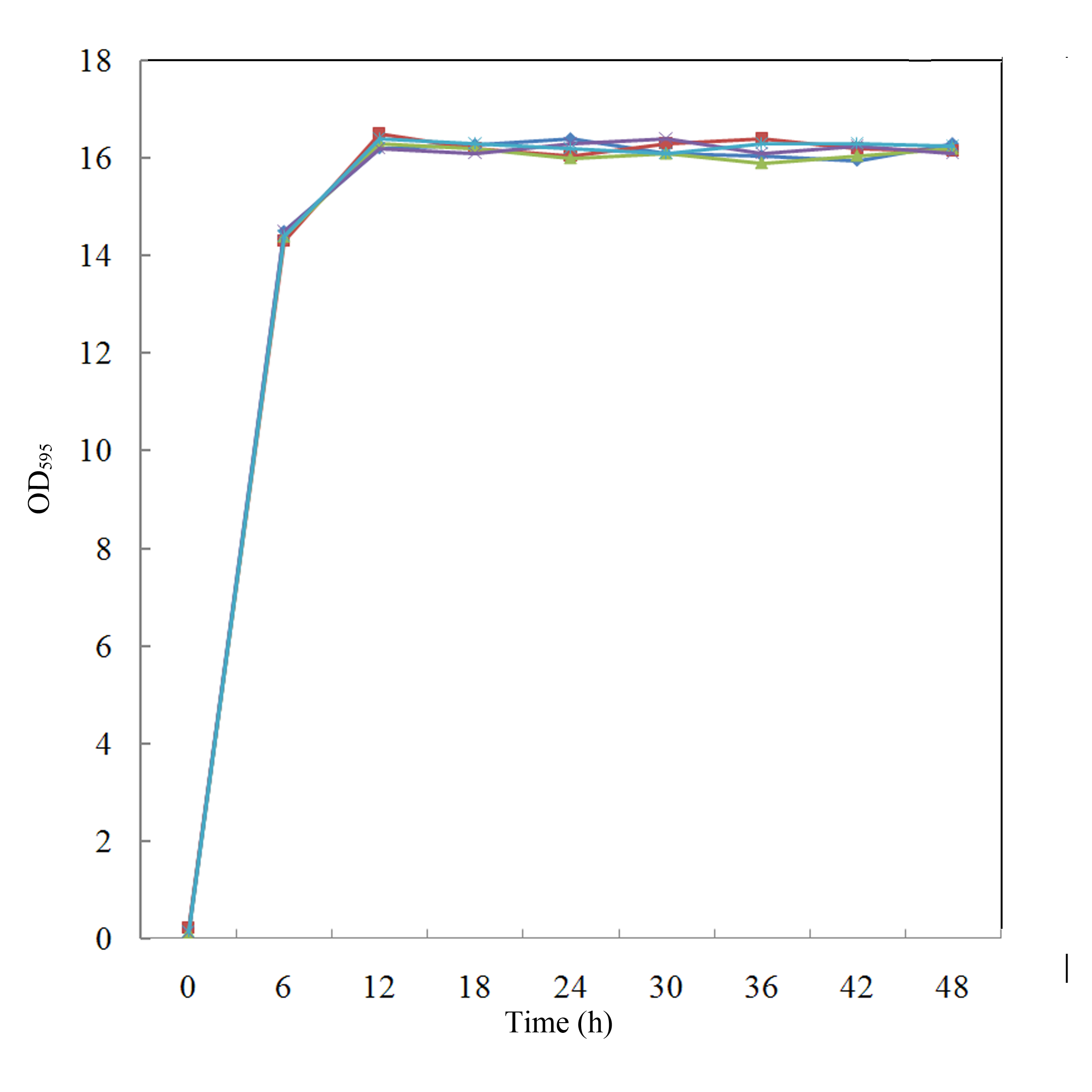

Supplement: Additional file 3 — The growth curves from B. subtilis 1A747 harboring different plasmids when cultured in LB supplemented with 5% maltose plus 5% glucose. (black diamond) represents OD595 from B. subtilis 1A747 harboring pLJ-7; (black square) represents OD595 from B. subtilis 1A747 harboring pJRINM1; (black triangle) represents OD595 from B. subtilis 1A747 harboring pJRINM2; cross (×) represents OD595 from B. subtilis 1A747 harboring pJRINM3; asterisk (*) represents OD595 from B. subtilis 1A747 harboring pJRINM4. [file 1475-2859-9-55-S3.TIFF]
