# Supplementary material for: Development of a Bacillus subtilis expression system using the improved Pglv promoter
Source: Microb Cell Fact. 2010 Jul 10;9:55. doi: 10.1186/1475-2859-9-55 (PMC2908567; doi:10.1186/1475-2859-9-55)
Supplement: Additional file 4 — Plasmids used in this study. [file 1475-2859-9-55-S4.DOC]

**Suppl. 4.** Plasmids used in this study

| Plasmids | Relevant genotype | Source or Reference |
| --- | --- | --- |
| pDL  pGJ103  pLJ-2  pBluskm  pJR1  pJR2  pJR3  pJR4  pLJ-7  pJRINM1  pJRINM2  pJRINM3  pJRINM4  pDG1728  pYG34  pB43  pCYL17  pCYL25 | *bgaB* gene donor  *E. coli*-*B. subtilis* shuttle vector  Promoter probe vector  Ap^R^, *E. coli* clone vector  pBluskm with P*_glv_*Ma  pBluskm with P*_glv_*Mb  pBluskm with P*_glv_*Mc  pBluskm with P*_glv_*Md  pLJ-2 with the wild promoter  pLJ-2 with the muted promoter inserted  in *Apa*I/*Eco*RI site  pLJ-2 with the muted promoter inserted  in *Apa*I/*Eco*RI site  pLJ-2 with the muted promoter inserted  in *Apa*I/*Eco*RI site  pLJ-2 with the muted promoter inserted  in *Apa*I/*Eco*RI site  specR donor  delivery vector  delivery vector  delivery vector  delivery vector | Yuan & Wong 1995  Yang et al. 2006  This study  Short et al. 1988  This study  This study  This study  This study  Yang et al. 2006  This study  This study  This study  This study  Guerot-Fleury et al. 1996  This study  This study  This study  This study |
