# Supplementary material for: Development of a Bacillus subtilis expression system using the improved Pglv promoter
Source: Microb Cell Fact. 2010 Jul 10;9:55. doi: 10.1186/1475-2859-9-55 (PMC2908567; doi:10.1186/1475-2859-9-55)
Supplement: Additional file 5 — Primers and oligonucletides used in this study. [file 1475-2859-9-55-S5.DOC]

**Suppl. 5.** Primers and oligonucletides used in this study

| Primer | Sequence (5’ →3’) | Restriction site |
| --- | --- | --- |
| Overlap-1-up  Overlap-1-down  Overlap-2-up  Overlap-2-down  Overlap-3-down  Overlap-4-up  Overlap-5-down  Overlap-6-up  Overlap-7-down  Overlap-8-up  P*_glv_*-1-up  P*_glv_*-5-down  INSD-1  INSD-2  bga-up  bga-down  GlvA-fro-up  GlvA-fro-down  GlvA-bac-up  GlvA-bac-down  Spec-I-up  Spec-I-down  P43-1-up  P43-1-down  B1-up  B1-down | TTTGCCAGCTCAATGACTTTATATGAGTCC  CGACCTCCTTGATAAATTTTACAATTCCATTTATAC  GTATAAATGGAATTGTAAAATTTATCAAGGAGGTCG  GATGTGCGCCATAACAAAATCGACATC  GATTTTTTCTTCATATGATCACCTCCTTGATAAATTTTAC  GTAAAATTTATCAAGGAGGTGATCATATGAAGAAAAAATC  CATATGACGACCTCCTTTCTAAATTTTACAATTCC  GGAATTGTAAAATTTAGAAAGGAGGTCGTCATATG  CATATGATCACCTCCTTTCTAAATTTTACAATTCC  GGAATTGTAAAATTTAGAAAGGAGGTGATCATATG  TTGGGCCCGGCATGTATCCGAATC  GCGGATCCTGGAGTGAAAGTGCTCCC  *G*ATCTTAAATAAATAAATTTATCAA*GG*A*GG*T*G*AATTC*G*A*G*CT  CGAATTCACCTCCTTGATAAATTTATTTATTTAA  GGCGAATTCATGAATGTGTTATCCTC  GCGGATCCAGGAGCTCTTAGC  TTGGTACCGTTTCCTGACACACCGTTC  TTGGGCCCTGAATTTTAAGTGAATATACGC  GCGGAATTCATGAAGAAAAAATCATTCTC  TTGAGCTCGGAATAATTGAGCATCC  TTGGATCCGAATGGCGATTTTC  GGCGTCGACTTGAAAAAAGTGTTTC  TTGGGCCCTCAGCATTATTGAGTG  GCGGAATTCATTCCTCTCTTACCTATAATG  AGAGTTTGATCCTGGCTCAC  AAGGAGGTGATCCAGCC | *Apa*I  *Bam*HI  *Eco*RI  *Eco*RI  *Bam*HI *Sac*I  *Kpn*I  *Apa*I  *Eco*RI  *Sac*I  *Bam*HI  *Sal*I  *Apa*I  *Eco*RI |
